# Right Ventricular-Pulmonary Artery Coupling in Transcatheter Tricuspid Repair: Metrics Under Scrutiny

**DOI:** 10.1016/j.shj.2026.100805

**Published:** 2026-01-29

**Authors:** Priscilla Wessly, Nadeen Faza

**Affiliations:** a Aurora Cardiovascular and Thoracic Services, Aurora Sinai/Aurora St. Luke’s Medical Centers, Aurora Health Care, Milwaukee, Wisconsin; b Department of Cardiovascular Medicine, Atrium Health Wake Forest Baptist Medical Center, 1 Medical Center Boulevard, Winston-Salem, North Carolina; c Department of Cardiology, Houston Methodist DeBakey Heart and Vascular Center, Houston, Texas

The management of severe tricuspid regurgitation (TR) has evolved rapidly with the advent of transcatheter tricuspid valve repair (TTVr) and replacement, providing less invasive alternatives for patients often deemed high-risk or inoperable for surgery.^1^^,^^2^ Chronic volume overload from severe TR progressively impairs right ventricular (RV) function, exacerbates pulmonary hypertension, and drives adverse outcomes. Accurate prognostic stratification is essential for optimal patient selection and intervention timing, yet reliable markers remain limited. RV-pulmonary artery (RV–PA) coupling—capturing the balance between RV contractility and afterload—has gained traction. The RV's myofiber architecture—subepicardial circumferential fibers continuous with the left ventricle and subendocardial longitudinal fibers (contributing ∼75% systolic function)—underpins its sensitivity to afterload, as seen in adaptive (concentric hypertrophy) vs. maladaptive remodeling (eccentric dilation, dyssynchrony) in chronic TR.^3^ This explains why coupling indices like tricuspid annular plane systolic excursion (TAPSE)/PA systolic pressure (PASP) remain robust in real-world TTVr populations with severe remodeling. More recently, RV free wall longitudinal strain (RVFWS)/PASP has been proposed as a potentially more sensitive, less load-dependent alternative, leveraging strain's ability to detect early myocardial impairment.^4^

In this issue of Structural Heart, von Stein et al.^5^ deliver a multicenter, retrospective analysis of 349 symptomatic patients undergoing TTVr (2017–2023) across three German centers. Patients were stratified by strain analysis vendor: GE EchoPAC (Vendor-A, n = 137) and TomTec (Vendor-B, n = 212). Procedures included tricuspid valve transcatheter edge-to-edge repair with the TriClip device (Abbott Vascularr, Abbott Park, Illinois) or the PASCAL device (Edwards Lifesciences, Irvine, California), or direct transcatheter tricuspid valve annuloplasty with Cardioband (Edwards Lifesciences), selected based on anatomy. The primary endpoint was a 2-year composite of all-cause mortality or first heart failure hospitalization.

Baseline differences were notable: Vendor-B patients were older, more symptomatic (New York Heart Association III/IV: 95.8 vs. 81.7%), had shorter 6-minute walk distances, and showed larger right heart dimensions despite comparable TR severity. RVFWS values were strikingly higher in Vendor-B (23.0 vs. 18.0%), underscoring vendor-related discrepancies.

The core findings reveal modest discriminatory performance for both indices (Harrell's C-statistics 0.564–0.646), with no significant superiority of RVFWS/PASP over TAPSE/PASP in either cohort. Multivariable Cox regression confirmed TAPSE/PASP as an independent predictor of the endpoint across both vendors, whereas RVFWS/PASP achieved independence only in Vendor-A, consistent with software-driven biases per Unlu et al.^6^ Receiver operating characteristics-derived cutoffs effectively stratified patients into subgroups with markedly divergent 2-year event-free survival. Sensitivity analyses, including procedure type and invasive PASP in a subgroup, affirmed robustness—although echocardiography underestimated PASP more substantially in the Vendor-B cohort.

These results highlight ongoing challenges in RV assessment for advanced TR. TAPSE/PASP's consistent prognostic independence aligns with prior evidence from Brener et al.^7^, who demonstrated in 444 TTVr patients that a baseline TAPSE/PASP ratio >0.406 mm/mmHg independently predicted lower 1-year mortality (adjusted hazard ratio 0.57, 95% CI 0.35–0.93, *p* = 0.023), outperforming TAPSE or PASP alone. This median value overlaps closely with von Stein et al.'s vendor-specific cutoffs. Patients with preserved coupling exhibited a postprocedural decline in the ratio—reflecting increased afterload from effective TR reduction—yet experienced better outcomes, highlighting "afterload reserve" in advanced TR. The concept of afterload reserve underscores the robustness of the simpler TAPSE/PASP index in advanced remodeling, whereas strain-based RVFWS/PASP is more vulnerable to vendor differences.

RVFWS/PASP, theoretically advantageous for its angle- and load-independence, showed promise in earlier TR^4^ cohorts but faltered here, especially in Vendor-B, where normal-range RVFWS values clashed with advanced disease phenotypes. This likely stems from intervendor bias driven primarily by software algorithms rather than image characteristics.^6^

Cohort disparities—Vendor-B with more severe phenotypes, exclusive transcatheter edge-to-edge repair use, and borderline higher massive/torrential TR (grade IV/V: 52.4 vs. 48.0%)—complicate direct comparisons. Proximal isovelocity surface area-based regurgitant volume assessment is notoriously challenging in massive TR, as echocardiography tends to underestimate effective regurgitant orifice area and volumes by 40%-50% due to nonhemispheric flow convergence zones, low-velocity jets, and tethered leaflets.^8^ This systematic underestimation likely contributes to the RV size paradox (larger dimensions in Vendor-B despite lower regurgitant volumes). Nevertheless, the modest C-statistics indicate that neither index fully captures post-TTVr risk, with 2-year event rates remaining high (34%–38%) despite TR reduction and symptomatic improvements. This underscores the need for multimodal prognostication incorporating biomarkers, invasive hemodynamics, and TR phenotypes (primary vs. atrial vs. ventricular).

In practice, TAPSE/PASP appears to be the more robust and vendor-independent metric, with relatively narrow and overlapping prognostic thresholds (0.367–0.442 mm/mmHg, closely aligned with Brener’s median of 0.406 mm/mmHg). In contrast, RVFWS/PASP shows substantial vendor variability (cutoffs 0.388%–0.583%/mmHg) and demonstrates prognostic value only within the GE platform (GE EchoPAC; GE Healthcare, Chicago, Illinois) cohort. Both indices offer only modest discrimination (C-statistics 0.56–0.65), so they should never be used in isolation. In advanced or massive TR, invasive PASP confirmation is strongly recommended to correct echo underestimation and improve risk stratification.

Current guidelines advocate early intervention in symptomatic severe TR before the onset of severe RV dysfunction or end-organ damage.^9^ Tools such as these may further sharpen shared decision-making, especially in patients who fall near the margins of procedural candidacy. The numerical improvement in discrimination with invasive PASP supports its use in equivocal or ambiguous cases, where echocardiographic estimates may be unreliable. As TTVr expands, prospective validation of vendor-agnostic strain thresholds will be essential to fully leverage its relative load-independence and to potentially extend its applicability to patients undergoing transcatheter tricuspid valve replacement. Emerging modalities—such as 3-dimensional echocardiography for RV volumes and function,^10^ cardiovascular magnetic resonance for fibrosis detection, or exercise echo for RV reserve—may overcome current limitations, enabling early intervention before irreversible RV remodeling.

We commend von Stein et al. for this pragmatic, vendor-aware analysis that reaffirms TAPSE/PASP as a reliable workhorse while exposing strain's current vulnerabilities in real-world practice. In the era of transcatheter tricuspid therapies, precision demands integration of advanced imaging to move beyond anatomic success toward sustained improvement in symptoms, better quality of life, and lasting clinical benefit.

## Funding

The authors have no funding to report.

## Disclosure Statement

The other authors had no conflicts to declare.

## References

[1] Sorajja P., Whisenant B., Hamid N. (2023). Transcatheter repair for patients with tricuspid regurgitation. N Engl J Med.

[2] Hahn R.T., Makkar R., Thourani V.H. (2025). Transcatheter valve replacement in severe tricuspid regurgitation. N Engl J Med.

[3] Lee J., Beneki E., Katsanakis N. (2025). Right ventricular dysfunction in structural tricuspid interventions. Eur Heart J Imaging Methods Pract.

[4] Ancona F., Margonato D., Menzà G. (2023). Ratio between right ventricular longitudinal strain and pulmonary arterial systolic pressure: a novel prognostic parameter in patients with severe tricuspid regurgitation. Int J Cardiol.

[5] von Stein J, von Stein P, Stolz L (2026). Comparison of echocardiographic right ventricular pulmonary-arterial coupling indices in patients undergoing transcatheter tricuspid valve repair. Struct Heart.

[6] Ünlü S., Mirea O., Bézy S. (2021). Inter-vendor variability in strain measurements depends on software rather than image characteristics. Int J Cardiovasc Imaging.

[7] Brener M.I., Lurz P., Hausleiter J. (2022). Right ventricular-pulmonary arterial coupling and afterload reserve in patients undergoing transcatheter tricuspid valve repair. J Am Coll Cardiol.

[8] Hahn R.T., Thomas J.D., Khalique O.K., Cavalcante J.L., Praz F., Zoghbi W.A. (2019). Imaging assessment of tricuspid regurgitation severity. JACC Cardiovasc Imaging.

[9] O'Gara P.T., Lindenfeld J., Hahn R.T. (2025). 10 issues for the clinician in tricuspid regurgitation evaluation and management: 2025 ACC expert consensus decision pathway: a report of the American College of Cardiology Solution Set Oversight Committee. J Am Coll Cardiol.

[10] Gavazzoni M., Badano L.P., Cascella A. (2023). Clinical value of a novel three-dimensional echocardiography-derived index of right ventricle-pulmonary artery coupling in tricuspid regurgitation. J Am Soc Echocardiogr.

